# Frequency of Switching Touching Mode Reflects Tactile Preference Judgment

**DOI:** 10.1038/s41598-020-59883-7

**Published:** 2020-02-20

**Authors:** Takumi Yokosaka, Masanobu Inubushi, Scinob Kuroki, Junji Watanabe

**Affiliations:** 1 NTT Communication Science Laboratories, Nippon Telegraph and Telephone Corporation, 3-1, Morinosato Wakamiya Atsugi-shi, Kanagawa, 243-0198 Japan; 2 Graduate School of Engineering Science, Osaka University, Osaka, 560-8531 Japan

**Keywords:** Sensorimotor processing, Human behaviour

## Abstract

We can judge affective aspects of objects by actively exploring them with our hands. Previous studies have mainly focused on how the physical properties of an object’s surface affect tactile preference evaluations. However, despite the widely accepted notion that the participant’s strategy has a great impact on how they explore an object, there is a lack of investigations of hand motion during preference judgment and its impact on preference rating. This paper recruits the recurrence plot technique to illustrate the temporal dynamics of explorative hand motion. In an experiment, participants were asked to freely explore the surface of tactile stimuli and rate their tactile preference for them. The temporal dynamics of finger velocity and force were visualized and characterized by using recurrence quantification analysis. We found correlations between preference ratings and recurrence features that represent the temporal dynamics of explorative hand motion, in addition to correlations between preference ratings and conventional time-averaged features (e.g., averaged finger velocity). One unique feature that correlated with preference ratings was TREND, which represents to what extent similar motion patterns repeatedly occur. The results of a subsidiary analysis supported the possibility that the TREND difference can be interpreted as the frequency of switching touching modes (e.g., stroking and pushing motions). Taken together, these results suggest that participants tend to perform the same hand motion repeatedly for preferable objects, while they tend to combine different touching modes for less preferable objects. They also indicate that the recurrence plot scheme is a promising way to extract humans’ strategies for tactile exploration.

## Introduction

We often touch and handle a product in stores to consider whether we like it or not. Investigating how we extract preference information through touch is important both for an understanding of the mechanism of human tactile perception and for designing attractive tactile products. Past studies highlighted the relationships between the physical properties of an object’s surface and tactile judgments related to preference. For example, participants tended to like surfaces with higher compliance^1^, while they tended to rate wet surfaces^2^ and those with a large dot spacing^3^ or large friction coefficient^4,5^ as disgusting or unpleasant. On the other hand, not only the physical properties of a touched surface but also hand motion during tactile exploration are closely linked to tactile perception, since inputs on the skin strongly depend on movement of the skin surface. Indeed, some studies showed that hand motion features such as averaged velocity, averaged force, and the peak value of force are correlated with evaluations of hardness, roughness, stickiness, and warmth^6–11^. However, the relationship between hand motion and preference evaluation for an object’s surface still remains unclear.

Explorative hand motion comprises a complex mixture of two effects: the effect of physical properties such as friction (bottom-up effect) and the effect of participants’ strategy (top-down effect). The top-down effect cannot be estimated by measuring the physical properties of an object’s surface, and, even when explorative hand motion is measured, it cannot be observed under experimental room conditions where participants’ touching mode is fixed (in this study, ‘touching mode’ is used to mean certain patterns of hand motion, such as stroking and pushing). Thus, few studies have been concerned with this effect. Nevertheless, the top-down effect is indeed strong as clearly evidenced by Lederman & Klatzky, who found that participants changed how they touched an object depending on what they wanted to judge^12^. In our previous studies^10,11^, we repeatedly measured explorative hand motion for the same set of texture stimuli by changing the participants’ task, i.e., the perceptual features to judge. Interestingly, we found that some ratings of perceptual features (e.g., stickiness) had a robust correlation with participants’ hand motion regardless of the task, while the correlation for some other features (e.g., warmth) depended on it. It can be speculated that for some perceptual features, the bottom-up effect is dominant in hand motion, while for others, the top-down effect is. Since preference, the main topic of this study, may reflect combinations of multiple perceptual aspects, it is highly likely that hand motion to judge preference will change as a result of both effects. We hypothesized that studying the strategy used (i.e., how an object is explored) to judge preference for an object’s surface will reveal how humans extract information about tactile preference. An earlier study, by showing that the averaged velocity of finger motion did not have a correlation with pleasantness ratings, suggested that there is no bottom-up effect on hand motion^4^. However, that study restricted participants’ touching mode to stroking only; therefore, the effect of the explorative strategy, i.e., the top-down effect, on preference judgment remains unknown.

Capturing the features of the touching mode from complex patterns of hand motion is challenging, since the touching mode category is not explicit in most cases and participants may use more than one mode. It is known that how participants touch an object is time-varying even within a single trial, where the tactile stimulus is not changed and physical properties are constant. For example, participants sequentially have used a couple of touching modes, such as grasping and stroking, to judge an object’s properties^13–16^. This view is also supported by a constructive approach showing that a robot arm could identify a tactile stimulus with higher accuracy by sequentially using multiple touching modes^17,18^. Other studies have shown that the averaged velocity and force tends to increase within a single trial^8,19^. Clearly, we need a better index than a categorical name or temporally averaged value to capture the temporal dynamics of hand motion.

Here, we introduce nonlinear time series analysis as an effective tool for understanding the temporal dynamics of explorative hand motion, and try to reveal the links between the observed temporal dynamics of unrestricted hand motion and preference rating. Nonlinear time series analysis is a well-established method often used for analyses of other kinds of explorative motion such as eye movement during scans of radiographs^20^ and wielding-hand motion during judgment of rod length^21^. One of the useful tools in the nonlinear time series analysis is the recurrence plot (RP) technique, which provides insight into the dynamics graphically^22^. Moreover, by calculating some characteristic values from RPs, such as DET, Lmax, and TREND as described below, we can quantitatively study the randomness, predictability, and stationarity of the time series, which is referred to as recurrence quantification analysis. Our main finding is that the index of stationarity (TREND) had a positive correlation with preference rating. We also found in a subsidiary analysis that recorded hand motions with smaller TREND contained switching of different touching modes. Taken together, these results suggest that human observers tend to change touching mode for unfavorable objects but perform similar hand movements for favorable ones. They also indicate that nonlinear time series analysis is a promising scheme for describing complex temporal dynamics of hand motion and understanding human tactile perception.

## Results

### Exp. 1: preference rating with 5-seconds free exploration

**Figure 1:**
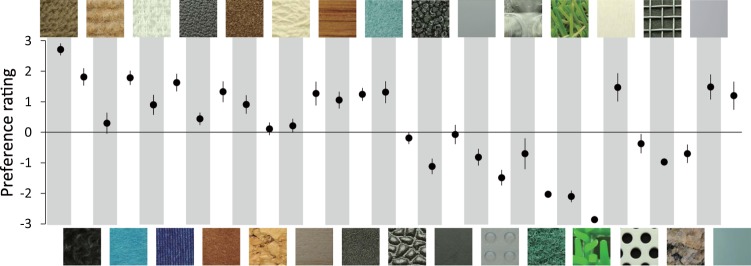
Preference ratings for 30 tactile stimuli (Fig. S1a). Error bars denote the standard errors.

In the experiment 1, participants were asked to touch one of 30 tactile texture stimuli (Fig. S1a) for five seconds and evaluate the preference level for each on a seven-point scale. Each of the 30 tactile stimuli was presented seven times (i.e., each participant performed 210 trials). Participants could use all fingers and palm of the right hand to explore the surfaces of the tactile stimuli and were not constrained in how to touch them. Visual information about the tactile stimuli was not available. The average ± standard deviation of reaction times in this experiment was 3.89 ± 0.17 seconds. The preference ratings made by participants are shown in Fig. 1.

### Recurrence features and the classification

**Figure 2:**
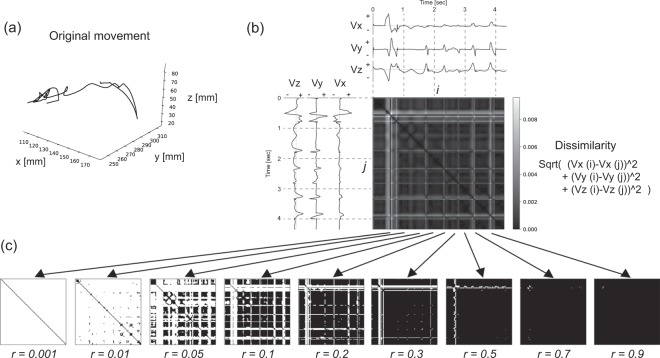
Examples of motion and RPs. (**a**) Finger motion in three-dimensional space. (**b**) Velocity profiles in each axis and two-dimensional image of the difference between $V(i)$ and $V(j)$, which is $d(V(i),V(j))$. (**c**) RPs $R(i,j)$ when threshold $r$ is varied. In this figure, recurrence points (points where $R(i,j)=1$) are plotted in black.

Index finger velocity $$V(t)=({V}_{x}(t),\,{V}_{y}(t),\,{V}_{z}(t))$$$$(t=1,2,\ldots T;{\rm{where}}\,T\,{\rm{is}}\,{\rm{the}}\,{\rm{length}}\,{\rm{of}}\,{\rm{the}}\,{\rm{time}}\,{\rm{series}})$$ and the force applied to a tactile stimulus $$F(t)=({F}_{x}(t),{F}_{y}(t),{F}_{z}(t))(t=1,2,\ldots T)$$ were measured and recorded during preference ratings. Here, we describe how recurrence features are extracted in the case of velocity data $$V(t)$$. An example of original movement is shown in Fig. 2a, and decomposed velocity trajectories $${V}_{x}(t),{V}_{y}(t),{V}_{z}(t)$$ are shown in Fig. 2b. The difference in the velocity at time $$i$$ and time $$j$$, which is represented as $$d(V(i),V(j))$$, can be calculated as the Euclidean distance between $$({V}_{x}(i),{V}_{y}(i),{V}_{z}(i))$$ and $$({V}_{x}(j),{V}_{y}(j),{V}_{z}(j))$$ in three-dimensional space. The differences in the velocities $$d(V(i),V(j))$$ for all combination of time sets $$(i,\,j)$$ can be drawn as a two-dimensional image as shown in Fig. 2b. In the recurrence analysis, this matrix is converted to a binarized two-dimensional matrix $$R(i,j)$$ according to a threshold $$\,r$$. If $$d(V(i),V(j))$$ is smaller than the threshold $$r$$, $$R(i,j)$$ is set to 1 to represent that the velocities $$V(i)$$ and $$V(j)$$ are similar. On the other hand, if $$d(V(i),V(j))$$ is larger than $$r$$, $$R(i,j)\,$$ is set to 0 to represent that they are not similar. RPs can be drawn by taking the points where $$R(i,j)=1$$ as black dots (recurrence points) and those where $$R(i,j)=0$$ as white dots. The pattern of RPs would be changed according to the threshold $$r$$ (Fig. 2c). Here, we adopted r = 0.1 since the fine structure of the images $$d(V(i),V(j))$$ (Fig. 2b) were kept on the RPs and the robust statistical correlations between preference ratings and recurrence features were observed (also see “Recurrence Features Related to Preference Judgment”).

**Figure 3:**
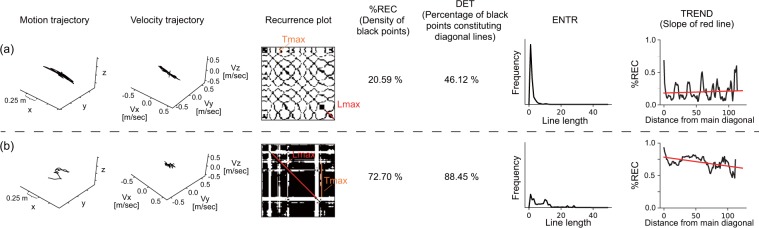
Examples of motion patterns, velocity trajectories, RPs, and visualized definitions of recurrence features. A participant performed a lateral stroking motion repeatedly in (**a**) and combined some explorative motions in (**b**). Since $\% REC$ at k = 0 is not used in the original definition of $TREND$, we plotted the k-$\% REC$ function with k = 0 discarded.

As a recurrence quantification analysis, we employ the following six statistical quantities: $\% REC$, $DET$, ${L}_{max}$, $ENTR$, $TREND$, and ${T}_{max}$^23^. First, $\% REC$ represents the percentage of recurrence (black) points in the RP. As stated above, recurrence points indicate that the velocities (or forces) between two time points are similar. Therefore, if participants perform finger motion with a constant velocity (or by applying a constant force), the RPs tend to have a lot of recurrence points, i.e., they have a large $\% REC$. If you look at the RPs of two different hand motion as shown in Fig. 3 for example, one RP (b) has more recurrence points than the other RP (a) and thus the $\% REC$ of the former is larger. Not only when the two time points $i$ and $j$ represent the same phase of repetitive hand movement but also when it accidentally moves at the same speed (or force), the difference in the velocity/force at time $i$ and time $j$ would be judged to be similar (i.e., to be a recurrence point) on an RP. It is essential to characterize the degree to which the dynamics behind the time series is deterministic or stochastic. $DET$ is defined as the percentage of recurrence points constituting the diagonal lines. When an RP has a lot of diagonal lines as in Fig. 3b, $DET$ tends to be larger. That is, large $DET$ means that many of the recurrence (black) points occurred because of the same phase of repetitive hand movement rather than by chance. $\,{L}_{max}$ is the length of the longest diagonal line excluding the main diagonal line (red line in the RP as shown in Fig. 3), which characterizes the predictability of the dynamics since a longer diagonal line means that similar motions were conducted for a longer period of time. $ENTR$ is the entropy of the probability distribution of line length (also see the histogram in Fig. 3), which reflects the complexity of the motion. The RP for more complex motion tends to have more variation in the length of the diagonal lines as shown in Fig. 3b. $TREND$ is defined as the regression coefficient for $\% REC$ as a function the distance to the main diagonal line (the slope of the red fitted line in Fig. 3), which reflects to what extent a motion changed over the time course (or statistical stationarity) and plays a central role in our study. If motion changes with passing time within a trial, the similarities of velocities/forces between two time points tend to decrease as the interval between the two time points increases (e.g., Fig. 3b). On the other hand, if the same motion pattern occurs repeatedly, the difference in motions would periodically change and can be small even when comparing two time points having large interval (e.g., Fig. 3a). The relationship between $\% REC$ and the distance to the main diagonal line and the slope of the fitted line can depict the global change (the gradual decrease or later increase) in similarity as shown in Fig. 3. ${T}_{max}$ is the length of the longest vertical line in an RP (orange line in the RP as shown in Fig. 3), which characterizes the longest time interval during which the motion is not active relatively. As shown in Fig. 2, the period having relatively small velocity tends to form a black area on the RP. Thus, a larger black area (i.e., larger ${T}_{max}$) indicates that relatively small motion is conducted for a longer time. Compared with the motion in Fig. 3a, the velocities of the motion in Fig. 3b are smaller, which results in larger ${T}_{max}$. In addition, to compare the recurrence features with conventional features, we calculated the average velocity and force across one trial as the feature $MEAN$. Thus, we calculated 14 features in total (i.e., $\% REC$, $DET$, ${L}_{max}$, $ENTR$, $TREND$, ${T}_{max}$, and $MEAN$ for velocity and force).

**Figure 4:**
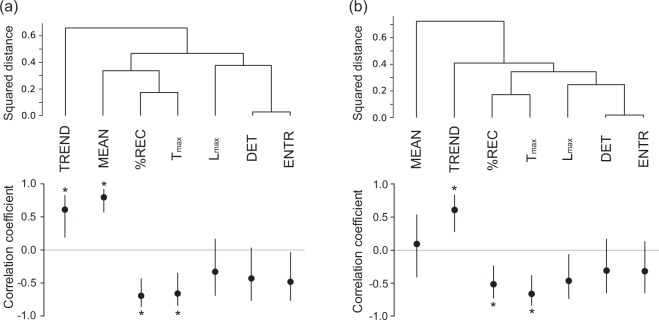
Results of cluster analysis for motion features (top) and Spearman’s correlation between motion features and preference ratings (bottom) for velocity (**a**) and force (**b**). Error bars denote the 95% confidence interval calculated by the bootstrap method^33^. Asterisks denote that the correlation coefficient was significantly different from 0 (see also Methods).

We conducted an agglomerative hierarchical clustering analysis with group averaging linkage to assess similarity between features. The results are shown in the top panels of Fig. 4. In these graphs, $DET$ and $ENTR$ are always neighboring each other in velocity and force, and the same is true for $\% REC$ and ${T}_{max}$, suggesting that these neighboring features resulted in a similar trend. Indeed, these features show a higher correlation with each other ($r$ = 0.89–0.99; Table S1a and b).

### Recurrence features related to preference judgment

**Figure 5:**
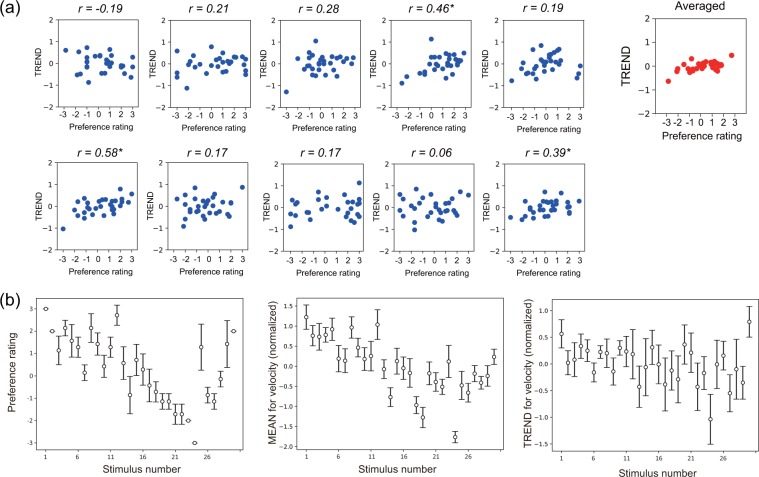
(**a**) Scatter plots between preference ratings and $TREND$s for each participant and for averaged data across all participants. The values of $TREND$s were normalized (average and standard deviation were 1 and 0, respectively) within each participant. The 30 blue dots in each panel represent 30 tactile stimuli, and seven repetitions were averaged within each stimulus. The 30 red dots represent the 30 tactile stimuli. Among the ten participants, one showed large (more than 0.5), two showed medium (0.3–0.5), and five showed small (0.1–0.3) correlations based on Cohen’s Guidelines^34^. (**b**) Examples of averages and standard deviations of preference ratings, $\,MEAN$s, and $TREND$s for velocity for each stimuli for the participant, who showed the largest correlation between $TREND$ and preference ratings.

**Figure 6:**
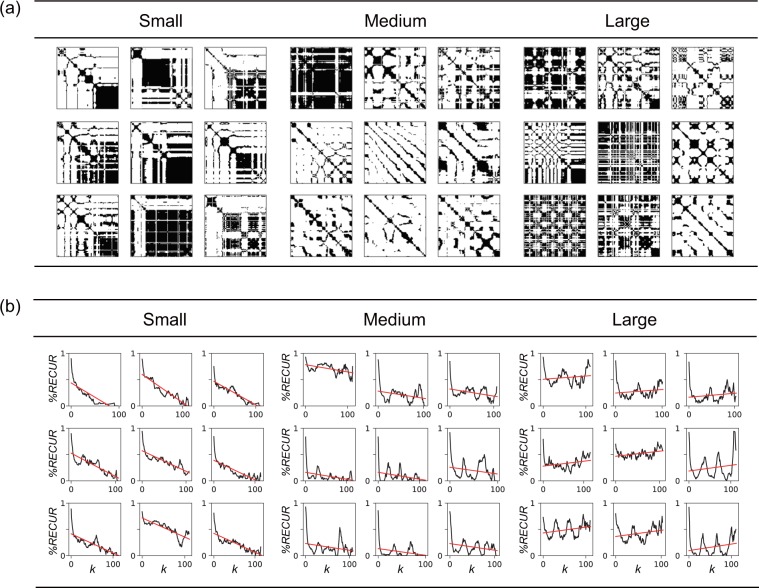
Examples of RPs of velocity grouped by $TREND$ value. Each RP was obtained with each trial for a single participant, whose absolute value of correlation between $TREND$ and preference ratings was highest. We sorted all 210 trials (30 tactile stimuli x 7 repetitions) based on the $TREND$ values and extracted the nine trials with the smallest $TREND$ (Small), nine with an intermediate $TREND$ (Medium), and nine with the largest $TREND$ (Large). (**a**) RPs of velocity for trials with the smallest, medium, and largest $TREND$. (**b**) Relationships between $\% REC$ and distance k from diagonal line for trials with the smallest, medium, and largest $TREND$. Red lines denote linear regression lines, whose slopes are known as $TREND$. The averages ± standard errors of preference ratings are 3.22 ± 0.44, 4.22 ± 0.52, and 4.44 ± 0.55 for smallest, medium, and largest $TREND$, respectively. Although one might consider that the first panel of the medium group appears to be an exceptional trial, $TREND$ by definition is unrelated to the density of recurrence points, and thus a dense RP could be grouped with relatively sparse RPs.

**Figure 7:**
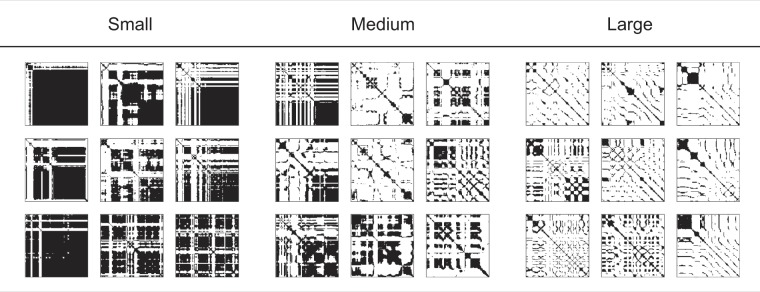
RPs of velocity for trials having smallest, medium, and largest $MEAN$ values. The averages ± standard errors of preference ratings are 2.67 ± 0.42, 4.00 ± 0.35, and 6.22 ± 0.31 for smallest, medium, and largest $MEAN$, respectively.

**Figure 8:**
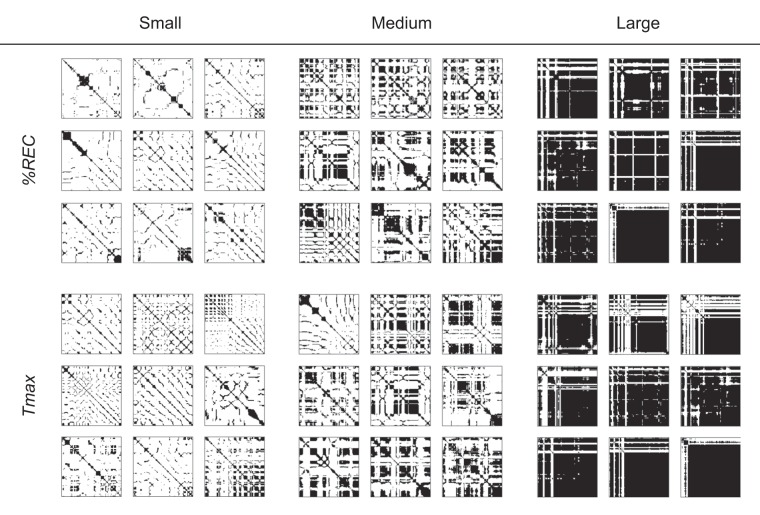
RPs of velocity for trials having the smallest, medium, and largest values of $\% REC$ and ${T}_{max}$.

Next, we computed Spearman’s correlation ${r}_{s}$ between features for velocity and preference ratings to find features related to preference evaluation (Fig. 4a, bottom). The preference ratings had positive correlations with $TREND$ and $MEAN$ and negative correlations with $\% REC$ and ${T}_{max}$. To gain further insight into the relationship between $TREND$ for velocity and preference ratings, we checked the velocity RPs of each participant. Though the results have some variation between individuals, almost all (nine of ten; also see Fig. 5) participants showed a positive correlation between $TREND$ and preference ratings. To save space, here we only show the RPs of one participant who showed the largest correlation to illustrate the relationships between $TREND$ and preference ratings. Figure 6a shows nine trials that had the smallest $TREND$, nine that had a medium $TREND$, and nine that the largest $\,TREND$ out of 210 trials. The RPs of the large $TREND$ group show finer and more uniform patterns, while those of the small $TREND$ group show more blocked patterns. Figure 6b shows fitted lines for the transition of $\% REC$ across distance from the main diagonal line (slope of the fitted line is $TREND$) for each panel in Fig. 6a. The $\% REC$s of large $TREND$ group show repetitive patterns of increases and decreases, while those of small $TREND$ group show monotonic decreases. The regular fluctuation of $\% REC\,$ for the large $TREND$ group might reflect the repetition of the same touching modes ($\% REC$ as a function of distance from the main diagonal line in Fig. 3a corresponds to this group). A similar trend was observed for other participants. Given the positive correlation with preference ratings, this finding supports the notion that participants repeated the same touching mode for more preferable stimuli but switched touching modes for less preferable stimuli. The positive correlation with $MEAN$ of velocity (the conventional feature) shows that, when touching less preferable stimulus, participants tended to move their fingers more slowly. Fig. 7 illustrates the RPs of one participant who showed a strong correlation between $MEAN$ and preference ratings; it consists of examples of small-$MEAN$ trials, medium-$MEAN$ trials, and large-$MEAN$ trials out of 210 trials. The RPs had more black areas in small-$MEAN$ trials, which shows that relatively small hand motion for longer periods of time results in larger black areas in the RPs. If hand motion were small for a longer time, $\% REC$ and ${T}_{max}$ would be large by definition. Thus, in contrast to $MEAN$, these two features would have negative correlations with preference ratings, which was observed (Fig. 8). Comparing Figs. 7 and 8, one can see that the categorization and patterns of RPs seem similar, though the direction of the effect is opposite (RPs with larger black areas appear in the group of large $\% REC$ and ${T}_{max}$, while similar RPs appear in the group with small $MEAN$ of velocity). Indeed, the $\% REC$ and ${T}_{max}$ strongly correlated with $MEAN$ (r = −0.84–0.90; Table S1).

**Figure 9:**
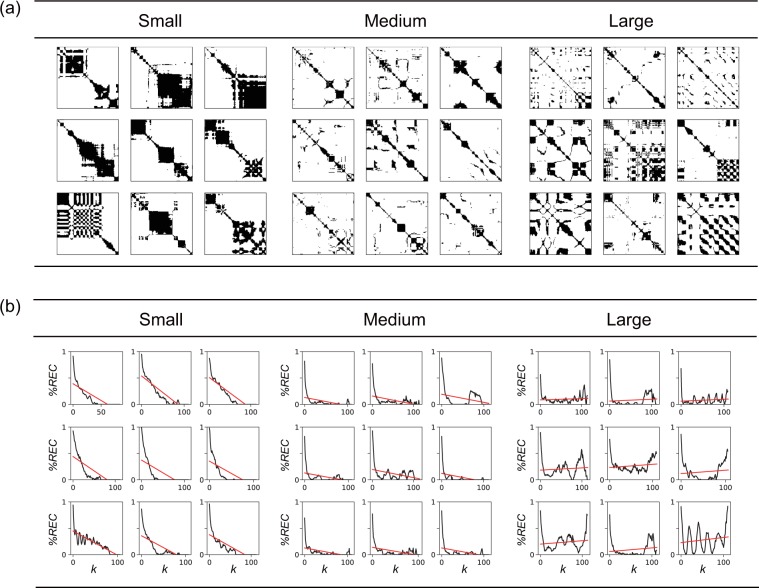
Examples of RPs of force grouped by $TREND$ value. Each RP was obtained with each trial for a single participant, whose absolute value of correlation between $TREND$ and preference ratings was highest. We sorted all 210 trials (30 tactile stimuli x 7 repetitions) based on the $TREND$ values and extracted nine trials with smallest $TREND$ (Small), nine with a medium $TREND$ (Medium), and nine with the largest $TREND$ (Large). (**a**) RPs of force for trials with the smallest, medium, and largest $TREND$. (**b**) Relationships between $\% REC$ and distance k from the main diagonal line for trials with the smallest, medium, largest $TREND$. Red lines denote regression lines (i.e., the slopes correspond to $TREND$). The averages  ±  standard errors of preference ratings are 3.22 ± 0.52, 3.11 ± 0.29, and 4.89 ± 0.55 for smallest, medium, and largest $TREND$, respectively.

**Figure 10:**
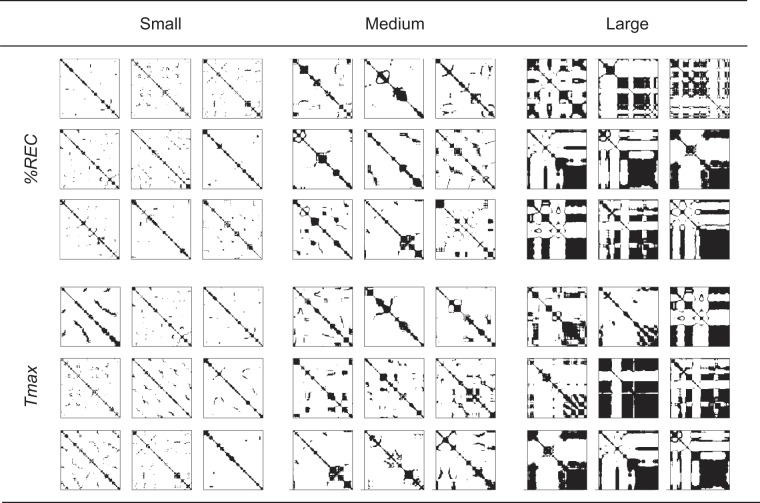
RPs of force for the trials with smallest, medium, and largest $\% REC$ and ${T}_{max}$.

We also assessed Spearman’s correlations between features for force and preference ratings (Fig. 4b, bottom). The ${\rm{MEAN}}$ of force did not have significant correlation with preference ratings. The $TREND$ positively correlated with preference ratings. To gain further insight into the correlation between $TREND$ and preference ratings, we checked the force RPs of each participant. Fig. 9a illustrates the RPs of one participant who showed a strong correlation, and the panels are grouped into smallest-$TREND$ trials, medium-$TREND$ trials, and largest-$TREND$ trials. Fitted lines for the transition of $\% REC$ across distance from the main diagonal line in each trial are shown in Fig. 9b. Like $TREND$ for velocity, the results show that the smaller $TREND$ group exhibits a blocked pattern, suggesting transitions between different motion patterns; while the larger $TREND$ group shows finer uniform patterns, though there are some exceptions. The $\% REC$ and ${T}_{max}$ for force are negatively correlated with preference ratings. The large $\% REC$ and ${T}_{max}$ group show large black areas (Fig. 10), which may reflect relatively small pushing motion. If this is the case, we can consider that participants tended to apply a small force to the surface for a long time when touching tactile stimuli rated as less favorable.

One might wonder if larger black areas in RPs reflects stops/pauses of hand motion. Here, to investigate how long participants stopped both stroking and pushing motions, we conducted an additional analysis. We created a new RP by ANDing the velocity RP and force RP and extracted the length of the longest vertical line on the new RP (Fig. S2a), which allows us to find black areas that were black (recurrence points) in both the velocity RP and force RP (i.e., we can speculate that participants did not perform both fast stroking and strong pushing in the black areas in the new RP). We refer to the length of the longest vertical line in the new RP as the ‘stopping period’. The histogram of the stopping periods is shown in Fig. S2b. Most of ‘stopping periods’ were shorter than 0.5 sec, and only 0.02% of all trials contained more than a 1.0-sec stopping period. From these results, we can consider that most of the large black areas indicate short stops for hand motion transitions (e.g., changing touching modes or the direction of their motion) rather than a longer pause of hand motion.

**Figure 11:**
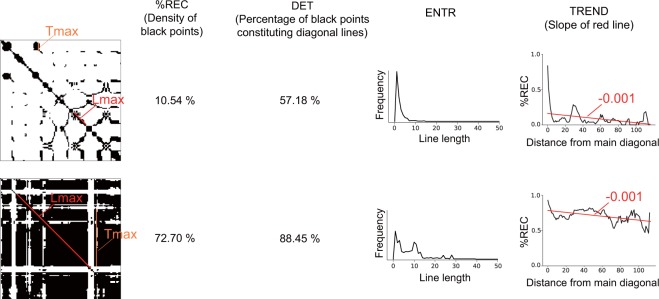
Schematic sketch of the effect of a larger black area in an RP on recurrence features. Except for $TREND$, the size of the black area affects recurrence features.

In summary, our results showed that $TREND$, ${\rm{MEAN}}$, $\% REC$, and ${T}_{max}$ for velocity and $\,TREND$, $\% REC$, and ${T}_{max}$ for force had correlations with preference ratings. It appears that these features can be divided into two major groups based on their tendencies. The $\% REC$ and ${T}_{max}$ tended to increase with increasing black area in RPs (Figs. 8 and 10), which is also true for $DET$, ${L}_{max}$, and $ENTR$. As illustrated in Fig. 11, this is because a larger black area (recurrence area) results in a higher density of recurrence points ($\% REC$), a higher density of diagonal lines ($DET$), a longer longest diagonal line (${L}_{max}$), a larger variation of diagonal line length ($ENTR$), and a longer longest vertical line (${T}_{max}$). The velocity $MEAN$ tended to decrease with increasing black area, too. The trials having larger black areas in RPs tended to consist of a slow motion phase and a fast motion phase (also see the example in Fig. 2b). The longer the slow motion phase is, the smaller the velocity $MEAN$ will be, only if the velocities during the fast motion phase are similar between trials. On the other hand, $TREND$ showed a unique feature in the sense that it can take almost the same value regardless of the densities of black areas, as shown in Fig. 11. Since past studies mainly focused on the effect of ${\rm{MEAN}}$ on preference ratings, here we discuss the other feature, $TREND$, in more detail.

### Subsidiary analysis: labeling touching mode switching by observing recorded motion

**Figure 12:**
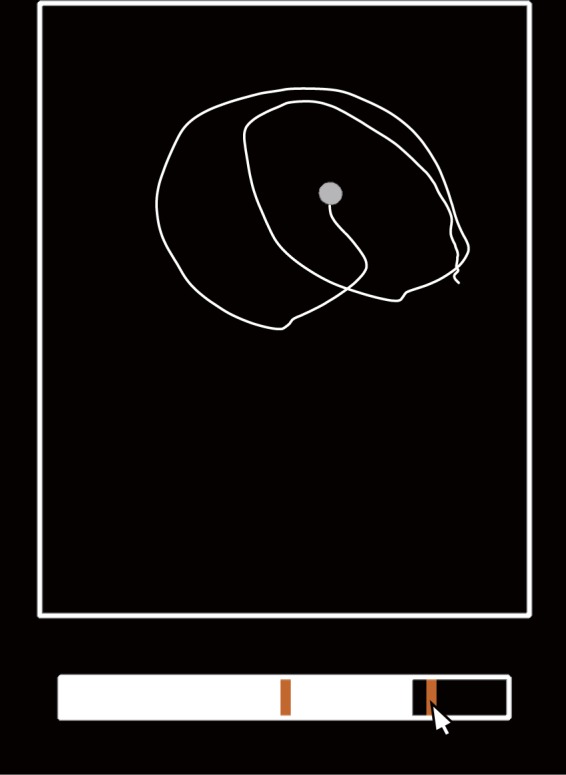
Example of visual stimuli used in the subsidiary analysis. Index finger movements recorded in the experiment 1 were shown as point-light motion. A white line showed movement trajectory, which was not rendered in the experiment. The horizontal bar below the point-light motion is a time gauge for the movie. Observers marked orange time points on the time gauge when they thought that the touching mode had changed.

The experiment 1 revealed that the recurrence feature $TREND$ can be a good index of preference rating, and it reflects quantitative changes in how participants touched (i.e., change in stationarity of hand motion). We observed that repeating the same touching mode (e.g., lateral stroking motion in Fig. 3a) results in larger $TREND$, but whether the larger $TREND$ can be attributed to participants’ use of the same touching mode remains unclear. To briefly check whether the trials with larger $TREND$ generally represent the same (or fewer switch of) touching mode, we conducted a subsidiary analysis where participants labeled the timing of the switching of touching modes by observing the measured motion. Point-light motions were rendered as stimuli, based on recorded finger motions in the experiment 1 (27 trials from Fig. 6a and 27 trials from Fig. 9a). The position of the index fingertip and applied force for a tactile stimulus were illustrated as the position and size of the point-light, respectively (Fig. 12 and supplementary movie). Ten observers who did not participate in the experiment 1 (i.e., they did not explore the stimuli) were asked to observe point-light motion and mark all the points in time where they thought the manner in which participants touched had changed.

**Figure 13:**
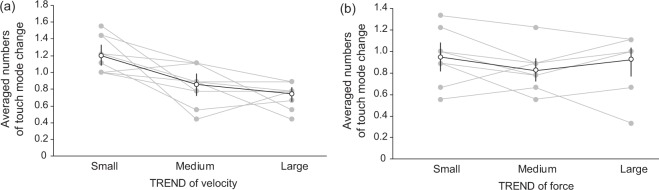
Results of subsidiary analysis. The open circles and line denote averaged results. Gray circles and lines denote the results for each participant. Error bars denote 95% confidence intervals calculated by bootstrap method.

The average numbers of reported touching mode changes across observers are illustrated in Fig. 13. The trials were divided into smallest, medium, and largest $TREND$ of velocity (Fig. 13a) and those of force (Fig. 13b). A one-way repeated measures ANOVA showed a significant effect of velocity $TREND$ (F(2, 27) = 15.44, ${\rm{p}} < 0.01,\,{\eta }^{2}=0.51$). Ryan’s method showed that the average number of motion changes in the trials with minimum $TREND$ was significantly larger than that in those with medium $TREND$ (${\rm{t}}(18)=4.03,\,{\rm{p}} < 0.01$) and with maximum $TREND$ (${\rm{t}}(18)=5.33,\,{\rm{p}} < 0.01$). On the other hand, a one-way repeated measures ANOVA showed no significant effect of force $TREND$ (${\rm{F}}(2,\,27)=15.44,\,{\rm{p}} < 0.01,\,{\eta }^{2}=0.51$). These results show that the observers tended to report more switching of the touching mode in trials with the smallest $TREND$ of velocity, whereas they tended not to report more switching in trials with the smallest $TREND$ of force.

### Exp. 2: preference rating with motion constraint

To see the top-down effect of explorative motion on the preference rating from other view point, we conducted an additional experiment wherein participants in the experiment 1 also judged preference for the same tactile stimuli set using only stroking motion or only pushing motion. We compared the preference ratings with those in the experiment 1 (since we did not constrain how participants touched the stimuli in the experiment 1, we refer to that as ‘free-touch condition’).

**Figure 14:**
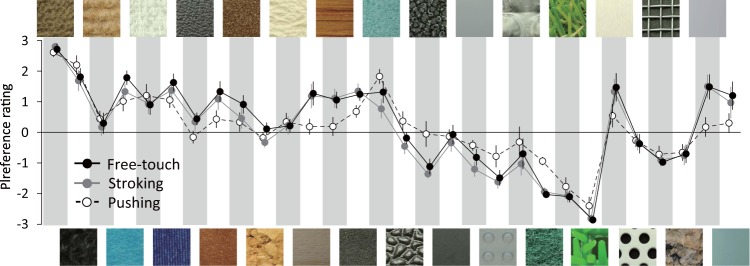
Preference ratings for each tactile stimulus in the free-touch condition (i.e., the results in the experiment 1), stroking condition and pushing condition. Error bars denote the standard errors.

We compared the preference ratings for each condition (free-touch condition, stroking condition, and pushing condition) to assess whether tactile preference is affected by touch motion. The results are shown in Fig. 14. A two-way repeated ANOVA with touch condition (free-touch, stroking, and pushing) and tactile stimulus as factors shows that there were significant effects of tactile stimuli (${\rm{F}}(29,261)=21.5$, ${\rm{p}} < 0.01$, ${\eta }^{2}=0.59$) and the interaction effect between the touch condition and tactile stimuli (${\rm{F}}(58,522)=5.22$, ${\rm{p}} < 0.01$, ${\eta }^{2}=0.04$), while the effect of the touch condition was not significant (${\rm{F}}(2,18)=2.94$, ${\rm{p}} < 0.08$, ${\eta }^{2} < 0.01$). A post-hoc comparison showed that the preference ratings in the stroking condition were identical to those in the free-touch condition, while those for some tactile stimuli in the pushing condition changed and tended to get close to a moderate rating. These results are consistent with the hypothesis that preference judgments can be affected by how a surface is explored.

### Exp. 3: preference rating without time constraint

**Figure 15:**
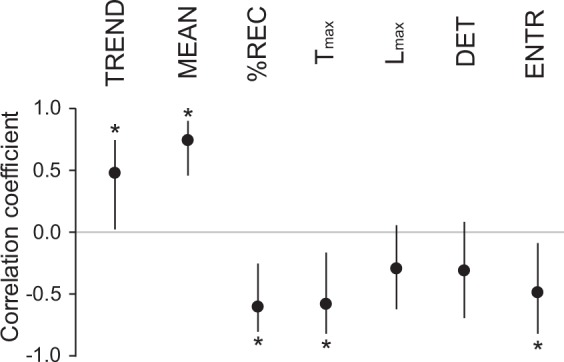
Results of Spearman’s correlation between motion features and preference ratings for velocity in the experiment without a time constraint. Error bars denote the 95% confidence interval calculated by the bootstrap method^33^. Asterisks denote that the correlation coefficient was significantly different from 0 (also see Methods).

One might suspect that the time constraint in our experiment (i.e., participants had to explore tactile stimuli for five seconds) resulted in the correlation between recurrence features and preference ratings. It can be speculated that participants performed a purposeless motion after they had decided their preference rating in their mind. Here, we repeated the experiment without a time constraint, where participants could stop explorative motion whenever they were ready to make a preference rating. The average  ±  standard deviation of reaction time in this experiment was 2.25  ±  0.58 sec. We computed the correlations between recurrence features for velocity and preference ratings (Fig. 15), which are in good agreement with the results of the experiment 1 (Fig. 4b). This indicates that observed correlations between recurrence features and preference ratings were not artifacts caused by our experimental paradigm (i.e., the constraint of exploration time). In addition, since eight of ten participants in this experiment did not participate in the experiment 1, the correlation between $TREND$ and preference ratings is robust to some extent for changes of participants.

## Discussion

In this study, we investigated whether hand motion features characterizing temporal dynamics have correlations with preference ratings. We found that not only averaged finger velocity within a trial (conventional finding) but also some recurrence features had correlations with preference ratings. In particular, $TREND$, which reflects to what extent similar recurrent patterns repeatedly occur, correlated with preference ratings in both velocity and force data. These correlations between the recurrence features and preference ratings were replicated even when participants made their preference ratings without a time constraint. The results of a subsidiary labelling analysis showed that the switching of touching mode was more obvious for observers in trials with small velocity $TREND$ than it was in trials with large $TREND$. Taken together, these results suggest that participants tend to keep on performing the same touching mode when touching more preferable stimuli, while they tend to switch touching modes when touching less preferable stimuli.

The correlation between averaged velocity and preference ratings shows that participants tended to move their finger faster on textures rated as more favorable. Both the bottom-up and top-down effect would be able to explain the correlation. In the context of the bottom-up effect, the reason for the correlation might be that textures having higher friction tend to be rated as less preferable^4^ and prevent participants’ smooth motion. On the other hand, in the context of the top-down effect, it can be speculated that participants tried to move their finger faster on the more preferable texture to get useful information or to fully enjoy it. In contrast to the conventional time-averaging feature reflecting a mixture of the two effects, $TREND$ is a more suitable index to represent the top-down effect since the stationarity/changing of the hand motion dynamics would mainly reflect participants’ strategy.

Switching touching modes for unfavorable stimuli might be explained by the analogous to research on ambiguous visual stimuli. It was reported that picture stimuli of ambiguous faces and food items were rated as less pleasant, possibly because these stimuli are difficult to categorize^24–27^. When this is also true for tactile domain, ambiguous tactile stimuli also make participants feel unfavorable toward them and would induce the use of combinations of different touching modes to accumulate more detailed information about them. In other words, switching of touching modes may reflect elaborate tactile exploration for ambiguous stimuli. One future direction to test this possibility is to measure stimulus identification performance in addition to preference ratings. Another possible reason for detailed exploration is that unfavorable stimuli were judged as dangerous. As participants could not see the tactile stimuli, they may have been concerned about the possibility of injury by the stimuli and thus carefully collected information about them. Not only the influence of less preferable stimuli but also that of more preferable stimuli can be considered. In our experiment, participants tended to repeat the same touching mode for a stimulus rated as more preferable at least for the length of tactile exploration in our experiment. It can be speculated that an optimal touching mode might make a touched stimulus more preferable (e.g., leather’s surface is preferable when rubbing it gently and a sponge’s surface is preferable when pushing it). Indeed, in the preference rating experiment during motion constraint tasks, we showed that preference ratings could be affected by how participants touched tactile stimuli, which might support this possibility. Investigating the optimal touching mode that makes each touched material more preferable will elucidate this possibility.

We also assessed whether the five-second constraint for the explorations in the experiment 1 resulted in the correlation between recurrence features and preference ratings because, if the five seconds was longer than participants’ judgment, participants might have touched a stimulus in a semi-systematic manner after they had readied to judge. Indeed, since the reaction time in the experiment without a time constraint (2.25 seconds) was shorter than that in the experiment 1 (3.89 seconds), our participants might have been able to judge tactile preferences earlier than the predetermined exploration time i.e., 5 sec. Nevertheless, the correlation pattern between recurrence features and preference ratings was very similar to that in the experiment 1. This finding indicates that observed correlations between recurrence features and preference ratings cannot be explained solely the five-second constraint. Participants would continue tactile exploration for preference judgment even after they made their ratings rather than stop meaningful exploration and touch in a semi-systematic manner for unpreferred stimuli.

One might argue that there is a possible discrepancy in $TREND$ (i.e., linear fitting) from the actual k-$\% REC$ data, due to the apparent fluctuation of $\% REC$ with changes in k (Fig. 6). Since $\% REC$ seems to exponentially decrease as a function of k in some trials, we tried to fit ${\rm{f}}({\rm{x}})={e}^{-\alpha x}$ to k-$\% REC$. Although the exponential decrease function has differing degrees of fitting (Fig. S3), the correlation between factor $\alpha$ and preference ratings was 0.74, which is larger than that between original $TREND$ and preference ratings (r = 0.61). Note that factor $\alpha$ might reflect the same type of quantity as mean velocity since it had a strong correlation with mean velocity (r = 0.94). Thus, we cannot simply conclude that factor $\alpha$ is an improved version of a (i.e., original $TREND$). Since there seems to be an initial steep change in $\% REC$ followed by a smooth decrease, the linear function may fit better by discarding $\% REC$ data around small k. We tested this possibility (Fig. S4) and found that although the fitting performance seems to be improved, the correlation with preference ratings dropped (Table S2). It can be speculated that the $\% REC$ data around small k play a role as a baseline to estimate to what extent $\% REC$ decreases with bigger k ($TREND$ was originally designed to extract this feature; Fig. S5), and this information is essential for estimation of the preference. Exploring a function that is able to explain the nonlinear relationship and to characterize the overall tendency would be needed in a future study.

Our study showed a correlation between preference ratings and time-averaged velocity, which was not observed in a previous study^4^. This apparent discrepancy can be explained by the difference in the objective of that study. The past study focused on stimuli’s frictional properties associated with pleasantness sensation. Participants’ explorative motion was restricted to lateral sliding motion of their index finger on a small stimulus plate. Therefore, the revealed relationship between pleasantness ratings and finger velocity mainly reflected the physical interaction between the finger and stimulus surface. In our study, on the other hand, participants’ motion was not restricted since our focus was on participants’ voluntary exploration strategy in addition to the physical interaction. Thus, the correlation between preference ratings and averaged velocity in our study would be based on participants’ voluntary strategy rather than physical interaction.

There were some differences between the velocity and force results. The preference ratings correlated with $MEAN$ of velocity but not with $MEAN$ of force. It is known that stroking motion, related to hand velocity, is useful for judging texture and that pushing motion, related to pushing force, is useful for judging hardness^6,7,12^. Exploring texture information might be deeply linked to preference judgment rather than hardness information. In addition, while both velocity $TREND$ and force $TREND$ had correlations with preference ratings, the subsidiary analysis showed more switching touching-mode with decreasing $TREND$ for velocity but not for force. This might result from difficulty in judging force switching. In our experiment, hand velocity was represented as the speed of point-light motion, while pushing force was represented as its size. It can be speculated that judging changes in force from changes in size was not intuitive, and thus our observers had difficulty in imagining the original force pattern. If this is the case, detection performance would be improved when pushing force is rendered in another way, such as a three-dimensional vector.

We treated the tip of the index finger as representative of the whole hand and measured and analyzed its position. We choose the index finger because it is known to be most often used for manual exploration, and the rest of the hand is less involved^28^. Still, the previous study focused on the judgment of basic tactile properties such as roughness and hardness, and little is known about how humans move their hands and fingers to judge tactile preference. Measuring and analyzing position data of other fingers or coordination between fingers might be useful for investigating humans’ exploration strategy for preference judgment.

In this study, we showed that nonlinear time series analysis is useful for visualizing and quantifying the temporal dynamics of explorative hand motion during preference judgment and can contribute to our understanding of the top-down effect (participants’ strategy). Through this analysis, we could quantify the dynamic temporal features (e.g., frequency of touching-mode switching), which had never been found in conventional time-averaging analysis. Though we could estimate switching between touching modes by using the analysis, each touching mode itself could not be identified since methods for identifying touching modes are still in progress^29–31^. Developing an identification method for each switched touching mode in a trial is warranted to analyse participants’ strategy in detail.

In summary, using nonlinear time series analysis, we investigated the links between the dynamic features of explorative hand motion and tactile preference judgment. We found that preference ratings correlated with $TREND$, ${\rm{MEAN}}$, $\% REC$, and ${T}_{max}$ for velocity of hand motion and with $\,TREND$, $\% REC$, and ${T}_{max}$ for force. The correlations with velocity ${\rm{MEAN}}$, $\% REC$, and ${T}_{max}$ would reflect that participants tended to perform relatively small hand motions longer when touching unfavorable stimuli. What is more, the positive correlation with $TREND$ (reflecting to what extent similar motion occurs again) suggests that we tend to change touching modes for an unfavorable object, while we touch a favorable one with a constant touching mode. Through observation of RPs and the subsidiary analysis, we found the possibility that the velocity $TREND$ can work as an index of the frequency of touching-mode switching within a trial, which suggests that $TREND$ may reflect participants’ strategy to extract information about tactile preference. Nonlinear time series analysis is a promising method to investigate human’s top-down strategy in tactile exploration.

## Methods

### Exp. 1: preference rating with 5-seconds free exploration

#### Participants

Ten naïve volunteers (five females) with an age range of 20–37 years participated in the experiment. All participants were right-handed and had normal or corrected-to-normal vision, and they had no known abnormalities of their tactile and motor systems. They had no specialized knowledge about psychophysical experiments and were unaware of the purpose of the experiments. They gave written informed consent before the experiment began. The experimental protocol was approved by the NTT Communication Science Laboratories Research Ethics Committee and was performed in accordance with ethical standards outlined by the Declaration of Helsinki.

#### Apparatus and stimuli

Participants sat at a table on which a tactile stimulus was placed. A black cover visually obscured the hand and tactile stimulus from the participants’ view. Thirty different materials, such as fabrics, leather, metals, and woods, were used as tactile stimuli. All the tactile stimuli were 30-mm thick and their surfaces were 100 × 100 mm squares. Index finger positions were recorded by tracking a retroreflective marker attached to the fingernail with four OptiTrack cameras (OptiTrack, Flex13, 120 Hz). Contact force on the tactile stimulus was recorded with a haptic force plate (Tech Gihan, 1000 Hz).

#### Procedure

Participants were instructed to freely explore the surface of a tactile stimulus to judge the preference level. They put their right index finger at the start position and started their exploration after a beep sound. Since it is known that participants tend to prefer a more familiar stimulus (longer exposure to a stimulus) not only in the visual domain but also in the tactile domain^32^, we fixed the duration of tactile exploration at five seconds to avoid the effect on participants’ judgment that might be caused by differences in explorative duration. When a second beep sound was presented after five seconds, they put their hand back at the start position and rated how much they preferred the tactile stimulus on a seven-point scale by pressing keys with their left hands. Each of the 30 tactile stimuli was presented in random order.

#### Analyses

To compute Spearman’s correlation, each feature and preference rating were averaged across repetition, standardized for each participant (i.e., the mean and standard deviation were zero and one, respectively), and averaged across participants (see also Fig. 5b for the variation of repetition for each stimuli). To judge whether a correlation between a motion feature and preference rating was above chance, we computed the confidence interval where the motion feature and preference rating had no correspondence. We randomly shuffled the order of the data and computed Spearman’s correlation coefficient for the data. After repeating the process ten thousand times, we assessed whether a certain range of the confidence interval included the original correlation coefficient. The ranges of the confidence interval were Bonferroni-corrected so that $p$ was 0.05 totally.

We resampled the finger velocity data and applied force at 30 Hz and calculated the RPs from the time series data without any state space reconstruction, since our focus was on understanding recurrence features based on the time series data. For RPs, we used finger velocity and applied force after participants had finished the reaching movement from the start position to the surface of tactile stimuli. The end time of the reaching movement was defined as the first point in time where the velocity of the index finger in the vertical direction became zero while the finger was on the surface of a tactile stimulus. The length of each trial was always five seconds, while the start point for analysing hand motion (i.e., the end time of reaching movement) depended on each trial; therefore, the data lengths for analysing hand motion varied across trials. For this reason, we normalized ${L}_{max}$ and ${T}_{max}$ by dividing the values by the data length for the hand motion analysis.

We conducted a cluster analysis to classify the 12 recurrence features and two time-averaging features. The distances matrix was calculated as a correlation matrix between the features subtracted from 1 (i.e., dissimilarity matrix). The correlations were calculated by using the standardized values (the mean and standard deviation were zero and one, respectively) of the features extracted from 300 conditions (10 participants × 30 tactile stimuli; seven repetitions were averaged).

The definitions of recurrence features are as follows.

- $\% REC=\frac{1}{{T}^{2}}\mathop{\sum }\limits_{t,s=1}^{T}\,R(t,s)$, where $T$ is the length of the time series.
- $DET=\frac{{\sum }_{l=2}^{T}l\,P(l)}{{\sum }_{l=1}^{T}lP(l)}$, where $P(l)$ is the probability distribution (histogram) of the lengths $l$ of the diagonal lines in RPs. If $P(l)=1$ when $l=\,{l}^{\text{'}}$ and $P(l)=0$ otherwise, all the lengths of the diagonal lines in RPs are $\,{l}^{\text{'}}$. On the other hand, if the distribution is flat, i.e. $P(l) \sim {\rm{const}}.$, there exist variations in the lengths of the diagonal lines in RPs. $DET$ represents the percentage of recurrence points constituting the diagonal lines. If the time series data are generated from a stochastic process, only single recurrence points ($l=1$) would be observed in RPs. On the other hand, if the time series data are generated from a deterministic dynamical system, we would observe not only single points but also many diagonal lines ($l > 2$) in RPs.
- $${L}_{max}=\,{\rm{\max }}\,\{l\,|P(l) > 0\}$$.
- $ENTR=-\mathop{\sum }\limits_{l=1}^{T}\,P(l)\log \,P(l)$, which is the entropy of the probability distribution $P(l)$. $ENTR$ is larger when the distribution is flatter.
- $TREND=\frac{{\sum }_{\tau =1}^{\tilde{T}}\,(\tau -\frac{\tilde{T}}{2})(R(\tau )-R(\tau ))}{{\sum }_{\tau =1}^{\tilde{T}}\,{(\tau -\frac{\tilde{T}}{2})}^{2}}$, where $R(\tau )=\frac{1}{T-\tau }\mathop{\sum }\limits_{t=1}^{T-\tau }\,R(t,\,t+\tau )$ is the recurrence rate in the line parallel to the diagonal of distance $\tau$ and $\tilde{T} < T$. Here, we use $T-T/10$ as $\tilde{T}$ since $T$ varied in each trial. If the linear relation $R(\tau )-\langle R(\tau )\rangle =a(\tau -\mathop{T}\limits^{ \sim }/2)$ holds, $TREND$ gives the slope, i.e., $TREND=a$.
- $${T}_{max}={\max }_{s}\{t|R(s,s+i)=1(i=1,2,\ldots ,t)\}$$.

Reaction time was defined as the time when a participants started to touch a tactile stimulus (i.e., end time of reaching movement) until the second beep signaling that five seconds had passed.

### Subsidiary analysis: labeling touching mode switching by observing recorded motion

#### Participants

Two of the authors (TY and SK) and eight volunteers (two females) with an age range of 22–36 years participated in the analysis. All participants had normal or corrected-to-normal vision. The eight volunteers were unaware of the purpose of the analysis. They gave written informed consent before the experiment began. The protocol was approved by the NTT Communication Science Laboratories Research Ethics Committee and was performed in accordance with ethical standards outlined by the Declaration of Helsinki.

#### Apparatus and stimuli

Visual stimuli were displayed on a computer screen (HP, ProDisplay P232, 1980 × 1080, 60 Hz). In the visual stimuli, the finger position and force of the participant whose $TREND$s showed the highest correlation with preference ratings were drawn. Finger positions were drawn as white circles by a three-dimensional rendering program from a top-down view. Force applied to tactile stimuli in three dimensions was converted to absolute distance from the origin and represented by the size of the white circles. A time bar was displayed under the finger motion (see Fig. 12). Each finger motion was repeatedly played until participants finished answering.

#### Procedure

Participants were asked to view finger motion created from the results of the experiment 1 and report the time points where finger motion changed by clicking the time bar. After participants had completed their answer, they could view the next finger motion. They could observe each finger motion as many times as desired until they finished their answers. A participant evaluated 54 finger motions (i.e., trials with smallest, medium, and largest $TREND$ shown in Fig. 6a and Fig. 9a). The presentation order of the finger motions was randomized.

### Exp. 2: preference rating with motion constraint

#### Participants

Ten volunteers in the experiment 1 also participated in this experiment. The other conditions were the same as in the experiment 1.

#### Procedure

The procedure in this additional experiment was the same as in the experiment 1 except that the participants were instructed to explore the surface of tactile stimulus with only stroking motion (stroking condition) or only pushing motion (pushing condition). We did not specify the speed of hand motion and the amount of contact force. These two conditions were rotated every 30 tactile stimuli and each tactile stimulus were evaluated seven times per condition.

### Exp. 3: preference rating without time constraint

The general procedure of this experiment was similar to that of the experiment 1. Differences from the experiment 1 were as follows.

#### Participants

Ten naïve volunteers (eight females) with an age range of 21–45 years participated in the experiment. Two participants in the experiment 1 also participated in this experiment. The other conditions were the same as in the experiment 1.

#### Procedure and analysis

Twenty-four different materials were used as tactile stimuli (Fig. S1b). Participants were instructed to freely explore the surface of a tactile stimulus and make the preference rating for the touched stimulus as soon as possible. Each of the 24 tactile stimuli was presented four times in random order. Reaction time was defined as the time when a participant started to touch a tactile stimulus until they made the preference rating by pressing a button.

## Supplementary information

Supplemental Movie.

Supplemental Materials.
