# Supplementary material for: Frequency of Switching Touching Mode Reflects Tactile Preference Judgment
Source: Sci Rep. 2020 Feb 20;10:3022. doi: 10.1038/s41598-020-59883-7 (PMC7033153; doi:10.1038/s41598-020-59883-7)
Supplement: Supplementary file 2 — Supplemental Materials. [file 41598_2020_59883_MOESM2_ESM.pdf]

Supplemental Material

Frequency of Switching Touching Mode Reflects Tactile Preference Judgment

Takumi Yokosaka*, Masanobu Inubushi, Scinob Kuroki, and Junji Watanabe

*To whom correspondence should be addressed. E-mail: yokosaka.takumi@gmail.com

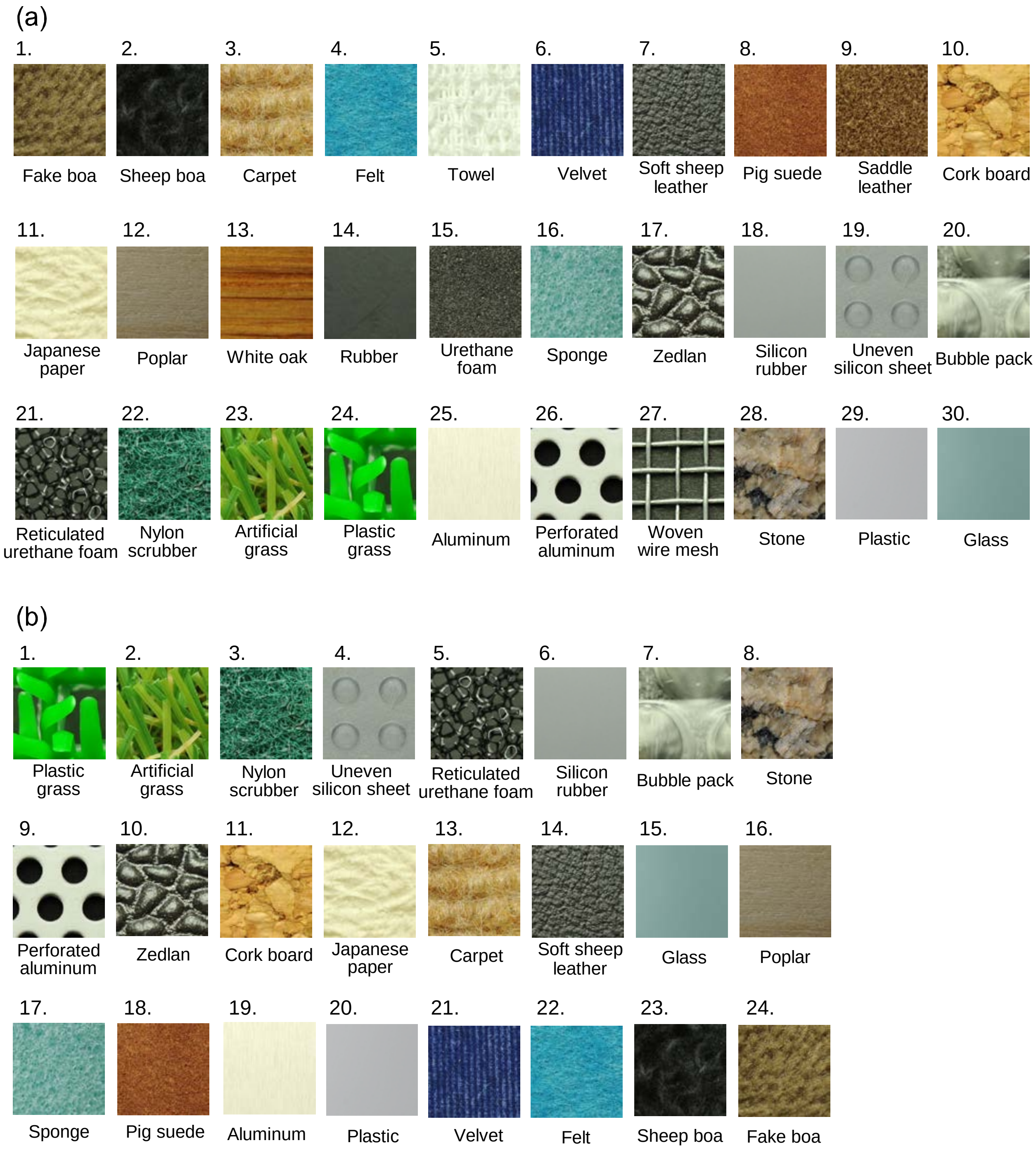

Figure S1. List of tactile stimuli used in the experiments 1 and 2 (a) and in the experiment 3 (b).

Table S1. Correlations between features for velocity (a) and force (b). Asterisks denote that the correlation coefficient was significantly different from 0 (also see Methods).

(a)

	DET	Lmax	ENTR	TREND	Tmax	MEAN
%REC	0.80*	0.55	0.84*	-0.55	0.95*	-0.90*
DET		0.83*	0.96*	-0.60*	0.87*	-0.77*
Lmax			0.76*	-0.73*	0.70*	-0.53
ENTR				-0.64*	0.88*	-0.79*
TREND					-0.66*	0.58*
Tmax						-0.84*

(b)

	DET	Lmax	ENTR	TREND	Tmax	MEAN
%REC	0.47	0.81*	0.48	-0.56*	0.89*	-0.09
DET		0.77*	0.99*	-0.65*	0.68*	0.64*
Lmax			0.77*	-0.71*	0.91*	0.25
ENTR				-0.62*	0.68*	0.66*
TREND					-0.78*	-0.07
Tmax						0.12

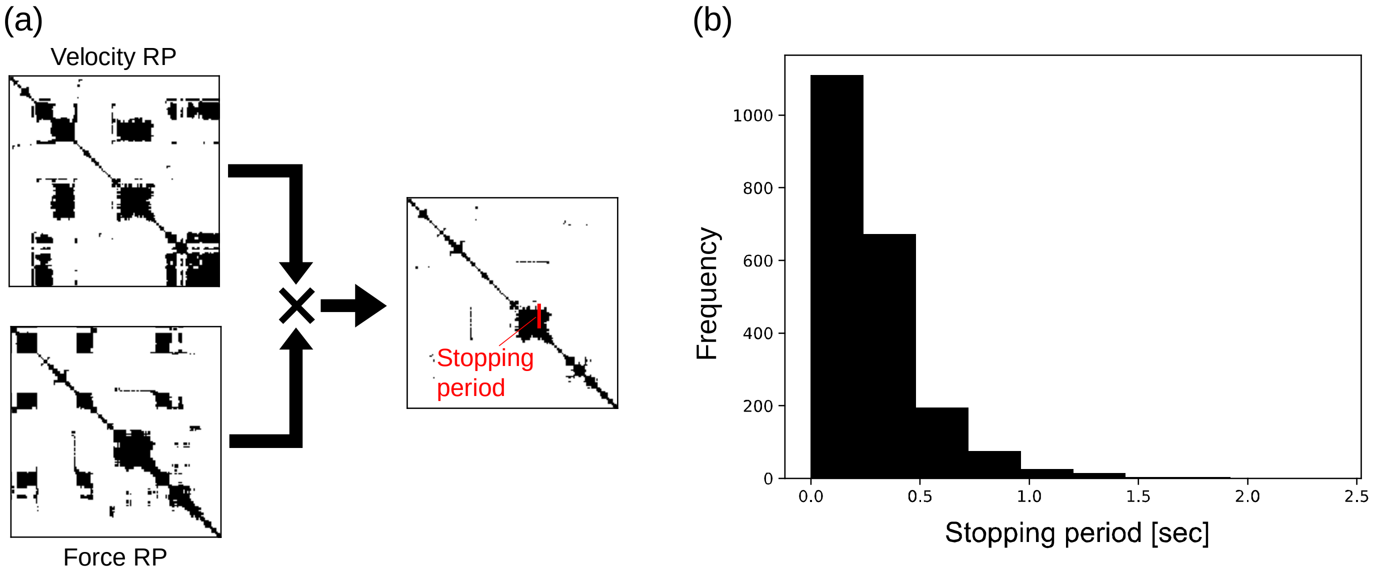

Figure S2. Analysis of stopping periods. (a) ANDing velocity RP and force RP allows us to find black areas that were black in both velocity RP and force RP. (b) Histogram of stopping periods for all trials (10 participants x 30 stimuli x 7 repetitions). Most of stopping periods were shorter than 0.5 sec, and only 0.02% of all trials contained more than a 1.0-sec stopping periods.

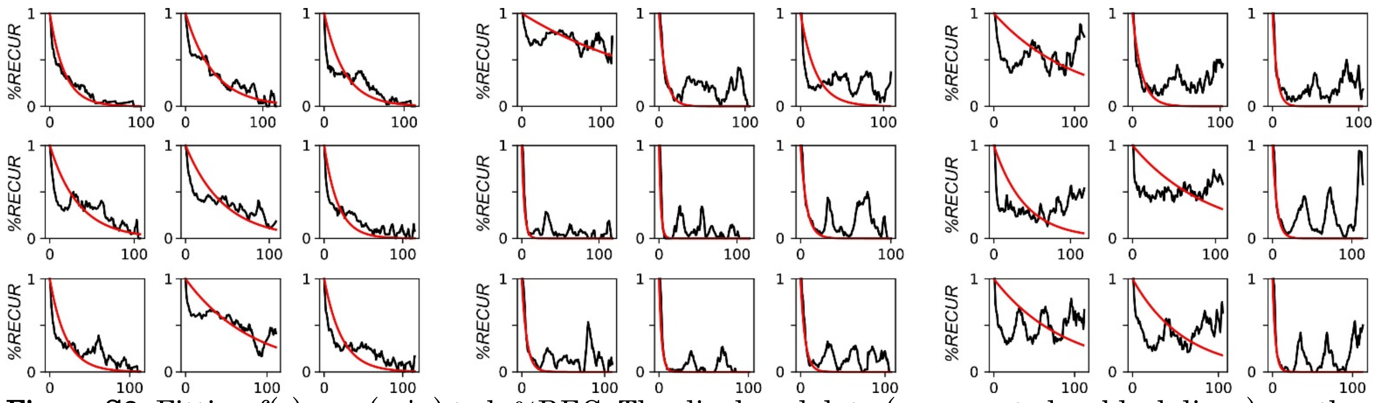

Figure S3. Fitting f(x)=exp(-a*x) to k-%REC. The displayed data (represented as black lines) are the same as in Fig. 6b, except that the data at k=0 are also plotted.

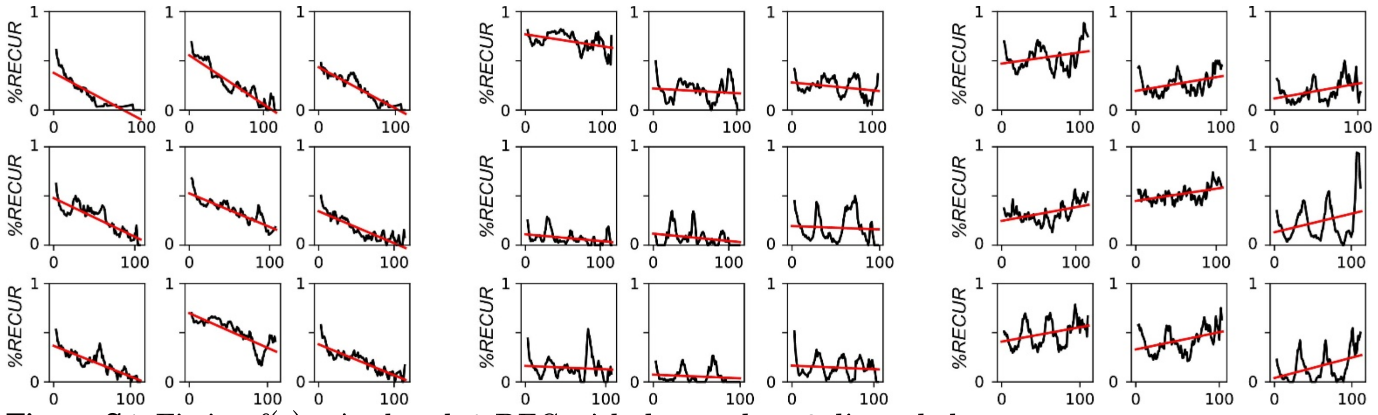

Figure S4. Fitting f(x)=a*x+b to k-%REC with data at k=1-3 discarded.

Table S2. Correlations between coefficient of fitted function and preference ratings with %REC data at small k discarded.

Original TREND	Discarding %REC data at k=1	k=1-2	k=1-3	k=1-4	k=1-5
0.61	0.59	0.57	0.55	0.53	0.52

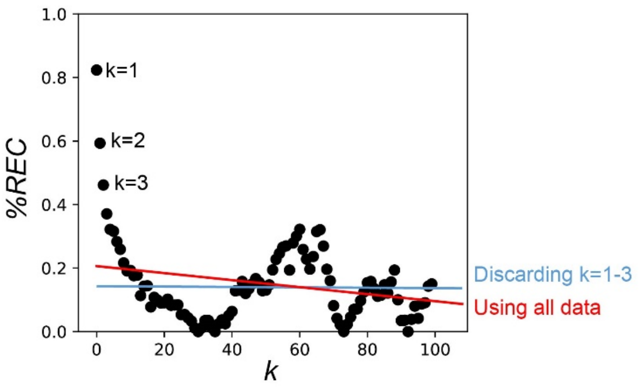

Figure S5. Difference in fitted lines with and without %REC data at k = 1-3. Discarding %REC data at k=1-3 makes it difficult to estimate the decreasing tendency.
